# Reasons behind current gender imbalances in senior global health roles and the practice and policy changes that can catalyze organizational change

**DOI:** 10.1017/gheg.2017.11

**Published:** 2017-12-10

**Authors:** C. Newman, P.K. Chama, M. Mugisha, C.W. Matsiko, V. Oketcho

**Affiliations:** 1 IntraHealth International Inc, Chapel Hill, North Carolina, USA; 2 Resource Mobilisation, Catholic Medical Mission Board, Lusaka, Zambia; 3 QD Consult Ltd., Kampala, Uganda; 4 MATSLINE Consult Ltd., Kampala, Uganda; 5 IntraHealth International Inc, Kampala, Uganda

**Keywords:** Gender discrimination, health care workers, organizations, policy and society, women's leadership, workplace

## Abstract

The paper distils results from a review of relevant literature and two gender analyses to highlight reasons for gender imbalances in senior roles in global health and ways to address them. Organizations, leadership, violence and discrimination, research and human resource management are all gendered. Supplementary materials from gender analyses in two African health organizations demonstrate how processes such as hiring, deployment and promotion, and interpersonal relations, are not ‘gender-neutral’ and that gendering processes shape privilege, status and opportunity in these health organizations. Organizational gender analysis, naming stereotypes, substantive equality principles, special measures and enabling conditions to dismantle gendered disadvantage can catalyze changes to improve women's ability to play senior global health roles in gendered organizations. Political strategies and synergies with autonomous feminist movements can increase women's full and effective participation and equal opportunities. The paper also presents organizational development actions to bring about more gender egalitarian global health organizations.

## Background

Achieving Sustainable Development Goal 5.5, which aims to ‘ensure women's full and effective participation and equal opportunities for leadership at all levels of decision-making in political, economic and public life’ [1] will depend on progress towards realizing all the targets for Sustainable Development Goal (SDG) 5. Improving the gender balance in senior global health roles in health research, policy, education and advocacy also depends on realizing other SDG 5 targets (see Table 1).

**Table 1.** SDG 5: Achieve gender equality and empower all women and girls

5.1 *End all forms of discrimination against all women and girls everywhere.*
5.2 *Eliminate all forms of violence against all women and girls* in the public and private spheres, including trafficking and sexual and other types of exploitation.
5.3 Eliminate all *harmful practices*, such as *child, early and forced marriage* and female genital mutilation.
5.4 *Recognize and value unpaid care and domestic work* through the provision of public services, infrastructure and social protection policies and the promotion of shared responsibility within the household and the family as nationally appropriate.
5.5 Ensure *women's full and effective participation and equal opportunities for leadership at all levels of decision-making* in political, economic and public life.
5.6 Ensure *universal access to sexual and reproductive health and reproductive rights* as agreed in accordance with the Programme of Action of the International Conference on Population and Development and the Beijing Platform for Action and the outcome documents of their review conferences.
5.7 Undertake reforms to give women *equal rights to economic resources*, as well as access to ownership and control over land and other forms of property, financial services, inheritance and natural resources, in accordance with national laws.
5.8 Enhance the *use of enabling technology,* in particular information and communications technology, to promote the empowerment of women.
5.9 Adopt and strengthen *sound policies and enforceable legislation* for the promotion of gender equality and the empowerment of all women and girls at all levels.

There are many reasons for these gender imbalances in global health and possible sites of change. This paper focuses on gender imbalances in senior global health roles in the context of health organizations, and the organizational inequality generating processes and mechanisms that abridge women's chances of being hired for a job, developing the requisite skills and knowledge to perform it, being fairly paid, enjoying equal treatment and advancing in a health career to senior leadership. It reviews factors that constrain women's full and effective participation and equal opportunities for ‘leadership at all levels of decision-making’ in research, policy, education and advocacy in health organizations. Many of these factors have already been documented in research or scholarship addressing barriers to women's leadership and workforce participation. This paper moves the field forward by offering a theoretical synthesis of barriers and shifting the analytical perspective from health systems, policy, programs, services, communities, to the health organization itself – not previously a focus of attention. The paper proposes four policies and practices to catalyse organizational changes, and a broader set of actions to bring about more gender-egalitarian (global) health organizations. It concludes by linking organizational change to gender equality movements in the larger society and in the global community. The reader is encouraged to review key definitions to support understanding of the concepts subsequently used in the paper, provided in Appendix 1.

## Methodology

The paper draws from a review of the literature and findings from two organizational gender analyses conducted between 2012 and 2014 in Zambian and Ugandan health organizations.

### Literature review

We conducted a literature review to examine key factors constraining women's full participation in and equal opportunities for varied roles and senior leadership in global health organizations. Several academic disciplines informed our work, including organizational studies, health workforce management, gender and development, gender and health, psychology, sociology, human resource and diversity management, human rights, and women's leadership. Specific databases and search terms are listed below:

1. Databases and sources included: APA PsycNET, MEDLINE, Project MUSE, Sociological Abstracts, Web of Science, Scopus, Political Science complete, United Nations Development Program (UNDP), United Nations Development Fund for Women (UNIFEM), Office of the United Nations Commissioner for Human Rights (OHCHR), PubMed Central, and Google Scholar.
2. Search terms included: Gender discrimination, gender inequality, workplace violence, wage discrimination, substantive inequality, occupational segregation, glass ceiling, pregnancy discrimination, family responsibilities discrimination, gender wage-gap, work- life reconciliation, gender and labor/employment rights, intersectionality, gender-based, sex-based, discrimination, workplace, environment, and harassment.

We also scanned stock-taking commentary and reviews in particular areas that were useful as syntheses of the state- of- the- field, as springboards to further reading, or that pointed to particular readings that were considered influential in terms of shaping the debate or turning the debate in new directions [For example, citations 2–5].

### Two organizational gender analyses

The authors also drew from the results of a mixed-method *gender discrimination and inequality analysis* approach conducted between 2012 and 2013 in a large public health system in Uganda and a small private-sector health system in Zambia. Methods included document review, employee and manager surveys, analysis of personnel records, focus group discussions, and key informant interviews. The results of the analysis of personnel records and focus groups provided particularly sharp insights into the gender composition and structure of jobs (i.e., gender segregation), and the inequality generating processes, which suggested reasons why men occupied the senior management and leadership rungs in both organizations. These results are presented in Appendix 2.

## Findings: Reasons behind the gender imbalance in senior roles

### Reasons

A recent review of interdisciplinary scholarship in women's leadership [2] from several fields suggested the reasons why, in general and across sectors (though not in global health), women do not ascend to senior organizational roles:

- Gender discrimination especially in the ways that leadership is defined to favor masculine-typed traits;
- Stereotyping, prejudice and perceptions of women's illegitimacy as leaders across racial/ethnic groups
- The lack of accumulated career capital;
- Group structures, composition and organizational contexts, such as the risky and prone-to-failure ‘glass cliff,’ in distressed organizations, the ‘Queen Bee’ phenomenon whereby women distance themselves from other women as a result of discrimination, or the level of group extraversion and decision-making procedures in women's emergence as leaders;
- The lack of availability of family-oriented work-life practices; and
- The lack of goal setting for higher female representation, such as targets, quotas and affirmative action/diversity reporting requirements.

This scholarship has also highlighted men's dominance of power and authority roles in organizations and in society, women's relative powerlessness in the face of sexual harassment and other forms of violence, and the lack of reproductive freedom [2].

Gender stereotyping and discrimination often have been implicated in preventing women from reaching positions of highest authority [6, 7]. This paper illustrates in more detail how cultural stereotypes, including the belief that women are not, or should not be, ‘agentic’ (i.e., denoting assertiveness, competitiveness, independence and mastery [8] and thus unsuited for leadership) are key constraints to women's senior representation in health, where it is estimated that women occupy only 25% of leadership roles in a sector where women make up 75% of the workforces in many countries [9, 10].

### The non-gender neutrality of organizations

Recent mobilization efforts have moved the issue of women in global leadership to the forefront of health systems advocacy [9]. To supplement global advocacy, more nuance and synthesis of evidence are now needed regarding the inequality-generating mechanisms and processes that constrain women's varied and senior roles in health organizations. This nuance is found in recent health workforce research in both employment and training systems, which sheds light on constellations of gender discrimination, including family responsibilities and pregnancy discrimination (together, ‘reproductive role’ discrimination), vertical and horizontal segregation, stereotyping and sexual harassment [3, 11–13]. More nuance can be found in scholarship and research into the larger organizational context itself – the structures, systems, cultures, leadership and power – in which these constellations of discrimination occur, as women participate in and lead, or alternatively, contend with marginalization or exclusion.

Health organizations are the products of gendered acts and processes that structure social relations in ways that are not distinct from the larger culture beyond its institutional walls [14]. This reproduction of social relations in organizations constrains women's roles and senior representation over the course of a career. There is by now substantial evidence for how organizations are gendered, masculinized [4, 14–25], and not ‘gender-neutral.’ Rather than being neutral, organizations are instead *inequality regimes* embedded in social structures and populated by people who bring their own (often non-egalitarian) cultures to work with them [16, 19].

Organizations are sites that actively construct and contest culturally dominant (hegemonic) masculinities and subordinate femininities [21], as well as subordinate and marginalized masculinities and ‘pariah (or deviant) femininities’ [26]. The ‘gender hegemony’ in an organization reflects the ideal gender order of a larger patriarchal culture [21, 26], in ways that produce distinctions between, and differently reward, masculine and feminine traits, thereby influencing male and female advantage, identity, power and control [17] in organizations.

Gendered organizational structure is manifest in the ways that work is designed, and in how work design creates conditions in which some workers who can comply with organizational work rules (typically men) and workers who cannot easily comply (typically women), resulting in problems of work-life reconciliation and in women's diminished ability to act on opportunities for participation and leadership [27–29]. Gendered conditions and organizational work rules to which men can more easily comply are illustrated in what have been called ‘maternal wall’ and ‘glass ceiling’ practices in organizations [28]. ‘*Maternal wall*’ practices include: Management using maternity as an excuse to not offer opportunities to mothers; passing mothers over for promotion; eliminating jobs during maternity leave or offering a demotion or less desirable assignments after childbirth and at return to work; the ‘executive schedule’ which requires overtime; marginalization of part-time workers; and expectations that workers who are ‘executive material’ will relocate their families in order to take a better job. ‘*Glass ceiling*’ practices include: Women's initial placement in relatively dead-end jobs; not getting job assignments that lead to advancement; not being promoted or closer scrutiny of women's performance relative to men's before promotion; and lack of access to informal networks and opportunities for mentoring. Results from the two African gender analyses in Appendix 2 illustrate some of these ‘maternal wall’ and ‘glass ceiling’ practices.

Organizational violence and coercion (such as sexual harassment, bullying, etc.) are also gendered [14] and serve to control and subordinate women and less dominant social groups in organizations [30]. A theoretician of patriarchy has remarked that ‘Male violence against women is sufficiently common and repetitive, with routinized consequences for women and routinized processing by judicial agencies, to constitute a social structure’ [31]. The extent to which violence is part of organizational structure is a subject for future global health research (see the discussion of sexual harassment, below).

If organizations are gendered, leadership is likewise gendered and how women are socialized to understand and enact the leader role brings all the stereotypes that come with gender roles into the gendered social space of an organization [32]. Examples of gendering processes and mechanisms in gendered organizations are given a closer look below, especially insofar as they contribute to gendered opportunity, disadvantage, privilege and the experience of leadership.

### A closer look at organizational gendering process and mechanisms

Consider the following processes and mechanisms that create and maintain the organizational ‘gender inequality regimes’ [19] that shape the types of work women can do, as well as the level of leadership women may attain, in global health organizations.

#### Gender segregation and stereotyping

Gender segregation is a pervasive and widely documented form of discrimination that creates rigidity in the types of jobs occupied by women and men in labor markets, in which women and men are expected to work in culturally defined, occupational roles dominated by their gender. It is one of the most enduring aspects of labor markets around the world [33]. Typically, women are *vertically segregated* and confined to a narrower range of work in marginal, lower-status and less well-paid jobs. Women often hold caring and nurturing occupations such as nurses, social workers and teachers and remain *horizontally segregated* from men, who are typically concentrated in technical, diagnostic, managerial, or strength-based jobs, as research scientists, physicians, managers, orderlies, etc.

Gender segregation is driven by cultural roles, employer and institutional bias, employee self- appraisal of the likelihood of success, preference, choice and labor commitment, access to networks, and especially by the gender stereotypes [34] that are embedded in policies, laws, traditional sayings, educational curricula and the media. The pervasiveness and intractability of gendered occupational structures are sustained by two deeply rooted tenets:

1. *Gender essentialism* posits that men and women have a basic unchanging ‘essence.’ Women are expected to be emotional, and more naturally competent in personal service, nurturance, and social interactions characterized by ‘niceness’ [34, 35] while men are believed to be more competent in tasks requiring leadership and rationality.
2. *Male primacy* represents men as naturally dominant and more status-worthy than women [34]. Male primacy underpins pro-male bias in hiring, compensation and promotion, and is predicated on gender status beliefs that men are not only ‘different than’ but ‘better than,’ or more worthy than, women.

Male primacy and gender essentialism define men and women as different in socially and occupationally significant ways [36]. By tying supposed innate traits to tasks, *gender essentialism* creates ‘occupational ghettos’ in organizations and labor markets that impede the crossover of men into female-identified jobs such as nursing, or vice versa [34]. Gender status beliefs that involve perceptions of women's lesser worth or inferiority as leaders act as barriers to women achieving positions of power and authority. For example, research in Rwanda found negative beliefs about the worth of female health workers, such that women ‘just don't know how to make decisions in a sure and certain way’ and that women ‘are not even capable of pulling out a tooth’ [11]. Beliefs that men have more worth (i.e., male primacy) act as facilitators to power, as well as barriers to their assuming positions of lesser social significance. For example, a belief in male primacy in Lesotho prevented men from crossing into the female-typed social role of caregiver, because it involved ‘free’ (volunteer) labor and low- status female-typed tasks, ultimately keeping men out of HIV/AIDS community-based caregiving and women almost exclusively in it [37].

### The glass ceiling as vertical gender segregation

The ‘glass ceiling’ can be understood as a form of vertical segregation, a mechanism underpinned by the inequality-generating process of stereotyping. The glass ceiling exists when typically unseen, artificial barriers that become more severe at higher occupational levels impede the advancement of women and minorities into top leadership [38]. The glass ceiling is based in part on the cultural association of the *agentic* manager trait with men, and implies a presumption of greater male competence in leadership [20, 36]. When women are perceived as equally assertive or masterful as men, they are often viewed as violating gender norms and essentialist beliefs that require women to be more communal or altruistic. When female managers act *agentically*, implicit gender biases lead others to react with resistance and hostility. Female leaders are often caught in a double bind, on the one hand experiencing disapproval if they display male-typed behaviors (such as asserting authority), while on the other hand, being negatively judged for female-typed behaviors, such as being supportive [8, 39, 40]. As a result, people frequently dislike highly competent women and question or reject their contributions and authority [7, 8]. Pariah femininities ‘contaminate’ a hegemonically ideal dominant/subordinate relationship between masculinity and femininity in the organization's gender regime, and are stigmatized as culturally deviant [26]. *Agentic* female managers who display ‘pariah femininity’ often face *backlash*, which also reinforces the glass ceiling. Stereotyping, the double bind and backlash are major contributors to women's under-representation at senior organizational levels.

#### Biased evaluations

Stereotyping perpetuates gender hierarchies by systematically over time biasing evaluations in ways that confirm beliefs about men's greater status and competence [20]. Biased evaluations play a major role in hiring and promotion decisions. Essentialist and male primacy stereotypes promote a competency bias against women interested in a leadership track or in roles that are ‘sex typed’ for men. For example, recent research illustrated how gender bias affects work-related appraisal of competence by describing a randomized, double-blind study that gave science faculty the application materials of a fictitious student randomly assigned a male or female name. The study found that both male and female faculty rated the male applicant as significantly more competent and hirable than the woman with identical application materials [41]. That is, gender bias led the raters to attribute greater competency to male applicants in a situation in which there were no objective differences. This research also found that biased assumptions about women's mathematical and scientific capabilities, expectations that female scientists act in ‘masculine’ ways in order to appear competent, and backlash for not acting in expected ‘feminine’ ways, pushed women out of science, technology, engineering and mathematics careers. A study of transgender men found that when one Barbara Barres, a professor who struggled to have her intellectual abilities taken seriously in undergraduate and graduate science courses became Ben Barres, his intellectual abilities and research were taken more seriously and given more value, epitomized by a colleague's remark, ‘Ben Barres gave a great seminar today, but then his work is much better than his sister's’. Barres concluded from his experiences that he was evaluated as a better scientist because he looked like a man [42].

Other research points to *relational* inequality-generating processes in organizations, such as the ‘opportunity hoarding,’ ‘resource pooling’ and ‘claims making’ about worthiness that resulted in men's greater organizational authority, respect, resources and rewards and thus, dominance in organizational cultures [16].

#### Stereotyping, pregnancy and family responsibilities discrimination

Gender beliefs and stereotypes foster workplace exclusions, restrictions, marginalization and inequalities particularly for pregnant women and mothers of dependent children, who face disadvantages in the labor force compared with men, and even compared with other women [43–47]. It has been said that ‘Motherhood is one of the key triggers for gender stereotyping’ [47], and indeed, pregnancy, motherhood and family have long been recognized as risk factors for unequal treatment at work [48–55], evident in the ‘maternal wall’ [51].

Stereotyping influences perceptions or expectations of pregnant employees’ and working mothers’ abilities, commitment, performance, and appropriateness for authority. Recent research [23] explored pregnancy-based discrimination, and identified processes of ‘symbolic vilification’ and ‘amplification’ in firing decisions. Pregnant workers were stigmatized through ‘*symbolic vilification’* of their competence and commitment that included charges of poor performance, proneness to absenteeism, unreliability and quitting. Regardless of the female employee's actual competence and commitment, pregnancy encouraged managers to *amplify* the ‘organizational good’ in order to legitimize their biases and justify dismissal or demotion. In this way, discriminatory treatment was passed off as a legitimate process in the service of reaching organizational goals [23].

These ultimately delegitimizing processes related to pregnancy also affect female employees who already have family responsibilities [52–55]. It is useful to view organizational exclusions and marginalization based on pregnancy and family responsibilities as related forms, encompassing a broad range of reproductive functions before, during and after childbirth, including childbearing, breastfeeding and ongoing child- and family caregiving. This may be viewed broadly as ‘reproductive role’ discrimination [13], which usually targets women of childbearing age who are not able to equally access opportunities for education, hiring, or promotion and experience breaks in the accumulation of career capital. ‘Reproductive role’ discrimination may also affect men to the extent that they prioritize family life in their working lives, making the sharing of responsibilities problematic (see the discussion of flexibility stigma in a later section).

#### The ideal worker

The ‘ideal worker’ is an organizational norm that structures organizational work, rewards and penalties by gender role. The term ‘ideal worker’ has appeared in discussions of work/life conflict since (at least) the late 1980s in anti-discrimination law debates, and later appears in sociological and occupational research [24, 25, 28, 56–59]. This norm operates in the male model of organizations [25]. An organization or workplace structured on the ‘ideal worker’ assumes that the worker can dedicate their lives to the job, with a related assumption that, if married, the worker is unencumbered by child-rearing or can depend on a wife to fulfill all, or nearly all, family responsibilities. Because employers often perceive pregnant women and working mothers as having divided loyalties between work and family life, they often assume that women lack the ‘ideal worker's’ commitment, and thus exclude women from consideration for positions structured for ‘ideal’ workers [58]. Beliefs that imply that individuals who are fully committed to work are naturally more suited to and more deserving of reward, responsibility and authority, while those with seemingly divided commitments belong in the lower ranks [24], are apparent in the male-typed ‘ideal worker’ norm [17, 24, 28, 56–59]. In the academic organizations studied in the US National Science Foundation's ADVANCE Institutional Transformation Program [25], the researchers found the ‘gendered organizational logics’ of the ideal worker underpinning the hierarchies, division of labor and ideas of how academic institutions should work. In these academic institutions, the ‘ideal worker’ was an achievement-oriented, unencumbered and competitive (male) research scientist. Women were disproportionately found in service and institutional housekeeping roles. Some women were able to successfully conform to the ‘ideal worker’ norm, which reinforced the legitimacy and desirability of these expectations in the study organizations [25]. While women's participation in paid work in organizations has changed considerably in the last 50 years, the male ‘ideal worker’ norm of full-time work is still implicit in how many workplaces and jobs are structured. This norm creates and maintains a divide between productive and reproductive activities, privileges wage over domestic labor [25, 59], and in situations of inadequate family-friendly support, fundamentally and practically disadvantages women, who are still disproportionately responsible for family responsibilities. In the end, women in particular still face an often subtle double bind: If a woman is an ideal worker, (how) can she be a good mother? And if a woman is a good mother, (how) can she be an ideal worker?

#### Sexual harassment

Sexual harassment is a form of gendered organizational violence [14] that severely constrains women's senior representation and productive participation in organizations. *Quid pro quo* (where an organizational superior makes favorable employment decisions conditional upon compliance with sexual demands), and hostile environment sexual harassment, result not only in abridgements of opportunity, but in personal, professional and economic harms, such as stress, leaving a job, transfer and demotion, all of which impact on the accumulation of career capital. The operation of gendered power to harass and subordinate is sometimes quite obvious, as when a female employee from one of the African organization's focus groups remarked, ‘*When men are bosses, they think they can take anything they want from female subordinates, so they start asking for sexual favors.*’ (see Appendix 2).

Another type, ‘power-threat’ sexual harassment, occurs when a person who has *greater* formal organizational power and authority is *targeted* for harassment by a person with lesser status [60]. It comes into play when women's higher standing in the organizational hierarchy is seen to challenge men's dominance in the gender regime regardless of institutional role. For example, in a study of gender and parliamentary politics in Uganda, the researcher [61] observed that women's sexuality was used as a means of reminding women of their sexual nature in a site of societal power and of their culturally subordinate status. Research from the USA found that cultures of sexual harassment created hostile environments that required the target's use of several adaptive strategies and that ultimately, sexual harassment was an effective means by which men were able to preserve more prestigious jobs [62]. Sexual harassment, which is driven by cultural and organizational norms that target women with non-professional, sexualizing and subordinating behaviors, is based on norms of masculine entitlement and feminine stereotypes ranging from sexual availability, provocation and acquiescence. This mechanism of organizational subordination makes women's work conditions and experiences substantially different from men's based on sex, and substantially disadvantaged in comparison with men's work conditions and experiences [58].

### Relevant results

Readers are again referred to relevant results in Appendix 2 from gender analyses conducted in two contemporary African health organizations. Together, the analysis of personnel data in conjunction with focus group data, illustrate some of the organizational gendering processes and mechanisms described above (e.g., the glass ceiling and ‘maternal wall’ practices). The analysis of personnel data reveals vertical segregation, an indicator of unequal opportunity. The focus group evidence suggests pro-male bias and a ‘discriminatory animus’ [58], where language illustrates relational, essentialist and male primacy beliefs pertaining to male and female health workers and leaders. Women's presumed emotionality, mood swings, tendency to make mistakes, lesser productivity, vengefulness, apparent inability to handle power, etc. compared unfavorably with men's superior mental agility, leadership, and versatility. Affirmative action was stigmatized as last resort of the unqualified. Health employment, work processes and interpersonal relations were not ‘gender-neutral’ in these organizations. Gender appeared to shape privilege and opportunity. Action plans demonstrate how these organizations used the results to pursue substantive equality policies and improve the gender balance in workforce participation and senior representation.

### Synthesis of reasons behind current gender imbalances in senior global health roles

This section has presented what research indicates to be the key contributors to gender imbalances in global health organizations, especially women's underrepresentation in senior roles. There is compelling evidence of the non-gender neutrality of organizations, that they are inequality regimes [19] involving active inequality generating mechanisms, which – like the gender hegemonic features of the larger (patriarchal) culture [21, 26] – legitimize male ascendancy and dominance and the subordination of women. The review also indicates that gender difference and relationality, privilege and disadvantage, are institutionalized in organizational structure and processes that include gender segregation, the ideal worker norm, glass ceiling and maternal wall practices, biased evaluation, reproductive role discrimination and sexual harassment, with reinforcing stereotypes that result in systemic structural discrimination against women (see key definitions in Appendix 1). It can be useful in future research, theory building and action to treat these processes and mechanisms as manifestations, or mutually reinforcing aspects, of gender segregation. The findings presented in this section have implications for the extent to which women will be able to attain the most senior representation in global health organizations without targeted and sustained change efforts. The health organization itself is not only the site of barriers, but of analysis and change.

## Practice and policy changes

The definitions in Appendix 1 clarify several ideas presented here and in Tables 2 and 3. Practice and policy changes to shift gender imbalances in leadership power and authority can take place within existing health organizations. Women can also find leadership opportunities in creating new health organizations. In this section, we consider the existing health organization as the site of new practices and policies. Our perspective is that some of the factors underlying gender imbalances in global health roles can be changed only if the non-gender neutrality of structures and culture is put in the forefront of organizational change efforts. Changing gender imbalances will require shifts in the principles underlying organizational policies and practices from formal equality to substantive equality, and from micro-level to macro-level change. It will require new perspectives in the ways the leadership is conceived and in how organizations and work are designed. In this section, we consider four practice and policy changes that can, to some extent, disrupt or dismantle organizational gendering mechanisms/processes, and reduce the systemic structural discrimination that figures so largely in the gender imbalance in senior global health roles.

**Table 2.** Four organizational practice and policy changes to catalyze changes in gender imbalances in senior global health roles

(1) Practice participatory organizational gender analysis to reveal organizational gendering processes, deep structures and culture; and ensure that this information is shared among institutional leaders, human resource managers and staff and used in organizational learning and human resource initiatives.
(2) Identify, name and raise awareness about the harms of *gender stereotyping* and implement strategies to eradicate them.
(3) Operationalize *substantive equality principles* in organizational governance and human resources management, particularly bearing on stereotyping, discrimination and work-life conflict.
(4) Put in place special measures and enabling conditions to promote substantive equality and dismantle the mechanisms that contribute to women's gendered disadvantage in gendered organizations.

**Table 3.** Principles, special measures and enabling conditions to promote substantive gender equality and dismantle gendered disadvantage in organizations

Substantive equality principles [66]	Special measures [66]	Enabling conditions [66]
**Affirmative mobilization:** Supporting, actively involving, building capacity to understand new measures and initiatives, raise awareness to claim rights and opportunities. [66]	Generate data for analysis, monitoring and evaluation. Name gender stereotypes [69, 73], competency bias, [19, 65] prejudices against female agentic leaders [4] [5]; strategies to eradicate stereotypes [73], explain how they harm human/ employment rights; analyze intersectional stereotypes Employee rights education that challenges stereotyping [66] Anti-discrimination advocacy to increase internal legal accountability for women's employment rights [99][57]	Strategies to deconstruct gender inequality regimes: Ways of organizing work other than hierarchy [87]; transformative/ feminist leadership models [88] Gender analysis of organizational systems, norms and gender regimes [20], structures of gender privilege/advantage [68], gendered reward and penalty systems (e.g., ideal worker, the wage penalty for motherhood [42], the “daddy bonus” [43]); and disincentives (e.g., flexibility stigma [83, 84]) Critical reflection and communications to identify and name stereotypes [73]
**Affirmative fairness:** Governance mechanisms and complaints procedures to address allegations of discrimination, and create disincentives against future discrimination [66]	Establish equal opportunity or gender equality mechanism [99] Promote diversity and nondiscrimination while preventing gender neutral policies that can negatively impact women [66, 75] Translate international human rights treaties and national laws into substantive gender equality and family- friendly HR policies [66, 99] Periodic wage evaluation to assure comparable worth [82] despite gender segregation [86] Zero tolerance policies for sexual harassment and education; enforcement to end impunity; employer liability Open-recruitment tools (e.g., public posting, employment agencies, hiring councils) to mitigate informal “old boy” social networks; job advertisements that target diverse applicants [102] Bureaucratic accountability in recruitment, hiring, and promotion [102] performance evaluation [4]	Dismantle the ideal worker norm [58]: Promote family-friendly workplaces and men's equal sharing of family responsibilities: modified time demands on workers; increased work flexibility; retrofitted workspaces for small children; incentivized maternity and paternity leave policies [26] Egalitarian systems through paid family leave policy provisions, working time regulations, and early childhood education and care [82]; clock stoppage on tenure track, automatic leave [24] Collective work redesign to dismantle the ideal worker norm [81]; avoid flexibility stigma and legitimize parenting/ caregiving responsibilities [27, 28, 50] Group relational strategies (e.g., mentoring programs to increase access to networks and role models [67] to supplement work redesign) Internal and external coalitions, collaboration and synergies with and external pressure from autonomous feminist movements [95], strategies to address political opposition and decrease segregation [94]; resistance strategies [96]
**Positive temporary measures:** Programs that actively seek out skilled women and minorities and place them in valued jobs, educational programs, and positions of authority in greater numbers than would otherwise occur [102]	Affirmative action [66] and priority recruitment, hiring and promotion objectives; setting targets and quotas [2] Assure critical mass of women in executive positions and work teams equal resource policies for male and female managers [102, 39, 4, 31] Dual hire programs [24]; appointment of women to high visibility and leadership tasks	Same as above.

The four practice and policy changes are:

- Practice participatory organizational gender analysis;
- Identify and name stereotypes and their harms;
- Operationalize substantive equality principles in organizational governance and human resources management policies; and
- Put in place special measures and enabling conditions to promote substantive equality and dismantle the mechanisms that contribute to women's gendered disadvantage in gendered organizations.

### Practice participatory organizational gender analysis

Participatory *organizational gender analyses* will reveal the gendering processes, mechanisms and structures of organizations. Wide participation in such critical analyses can build collective capacities to reflect on, and ultimately challenge the structures and norms of discrimination and inequality that impede the realization of individual and organizational potentials. It is essential to analyze the structures, processes, work culture and use of power in which ‘hegemonically defined masculinities and femininities’ play into ‘the constitution, reproduction and allocation’ of organizational power and meaning and rewards, that is to say, the institutional gender regime [19, 21, 25]. Institutional governance leaders, human resources managers and employees should examine: The organizational *gender regime* and its masculinities and femininities; maternal wall and glass ceiling practices; the composition of jobs and hierarchical positioning of the organization's workforce by gender and other axes of exclusion; formal and informal organizational rules, authority and power centers; leadership models; work design and arrangements for work-life reconciliation; perceptions of opportunity, bias and forms of discrimination and violence (e.g., sexual harassment or other forms of organizational violation [14]); gender stereotypes including those related to reproductive roles, science and leadership for men and women; and policies that promote equality and nondiscrimination. Gender analysis data should be used for organizational learning, shared as widely as possible and used in employee education and human resources initiatives.

While gender relations of power constitute the root causes of gender inequality in- and outside organizations, gender *intersects* with class, racial, ethnic, caste, religious and other markers to create different social identities, hierarchies and opportunities and disadvantages [63–67]. To be effective and relevant, these dimensions of identity and social status should be incorporated into organizational gender analyses, not to mention the organization's health research, policy and advocacy initiatives (see Appendix 3: Analysis of Gender and Intersecting Inequalities, which provides relevant articles with guidance on how to understand intersectional analyses in health research, and offers thoughts on an organizational gender analysis approach that addresses intersectional inequalities).

#### Levels of gender analysis and action

Since organizations, groups and individuals are influenced by the ‘mega’ level of society, mega- level factors such as social evolution and political, economic and sectoral developments outside of the organization should be monitored and taken into account in gender analysis and action.

We should be also interested in the ‘micro’ level of the individuals who work in global health organizations, including their beliefs, attitudes, biases and interactions. However, gender analysis should have an organizational (or macro) focus and subsequent action must target organizational change. We should therefore be skeptical of the long-term effectiveness of only or mainly micro-level change interventions [25]. For example, mentoring and access to professional networks have had meaningful effects on the motivation, confidence and self-efficacy of female researchers through exchanges with role models and peer support [68]. Such valuable interpersonal interactions and the expansion of networking and mentoring opportunities for individual women are certainly welcome antidotes to the social exclusion and isolation that affect women in male dominated contexts [25]. However, interventions that focus on opportunities for women are not long-term solutions to the organizational gendering processes, mechanisms and structures detailed earlier, such as the organizational norm of the ideal worker, with its institutional distinction between (valued) productive and (not valued) reproductive activities. As meaningful as they may be, individual or micro-level change strategies usually only help women integrate into, and be more successful in, masculinized organizational cultures [25]. This limitation also applies to individually negotiated flexible work-family arrangements [25, 29]. In the end, changing inequalities in opportunity and access in organizational systems must be addressed in changes at the ‘*macro’* (organizational) level, through the dynamic relationship between individuals and organizational systems [69].

This is to say, while it is reasonable to think that change strategies should in some way target individuals in organizations (since social change is ultimately a matter of people's transformed behavior), the key to sustainable change in the gender imbalances in global health organizations will be to connect individuals to the organizational systems in which individuals participate [69]. It starts by engaging in critical (gender) analysis of organizational systems themselves and especially, how employees participate in organizations in ways that either reinforce or challenge the systems and cultures that impede gender equality [69]. One example of this would be to reflect critically on the nature and extent of hegemonic masculinity and emphasized femininity in the organization, such as men's and women's collusion in the unequal distribution of power or the extent of women's compliance with the unequal structuring of gender relations, in situations where non-compliance matters for women's leadership opportunities. The gender regime may be undone by changing how we participate in systems, because our participation makes the system ‘happen differently’ [69].

### Identify, name and raise awareness about the harms of gender stereotyping and implement strategies to eradicate them

Critical analysis necessarily includes analysis of organizational and cultural gender stereotypes. Stereotypes are typically relational in nature [26] and assign women and men distinct yet mutually reinforcing attributes, characteristics or roles, which have by now obvious career consequences. For example, the relational stereotypes in the FGD narratives in Appendix 2 convey that men need help in controlling their (sexually harassing) behavior in the face of women's provocativeness, while at the same time, convey men as rational, competent and reliable leaders, in contrast to women, who must demonstrate their competence because they are psychologically unfit for management, incompetent, vengeful, emotional, preoccupied or moody, unable to wield power wisely, or unreliable on account of uncontrolled fertility. Such relational stereotyping kept women out of leadership jobs. Changing these stereotypes would require fundamental changes in mindset and gender relations in society, beyond the scope of organizational change. But the first steps in increasing the number of women in more varied and senior roles require challenging the stereotypes that uphold vertical segregation in organizations.

#### The human rights challenge to gender stereotyping and strategies to eradicate it

Human rights law concerns itself with gender stereotyping because it violates recognized human rights and fundamental freedoms, such as the right to equal opportunity and nondiscrimination, the right to fair hiring and career progression, the right to decent work and the right to bodily integrity [70, 71]. Stereotypes can be hostile/negative (e.g., women are emotional) or seemingly benign (e.g., women are nurturing), but in the end, they are harmful. Intersectional gender stereotypes have a disproportionate negative impact on certain groups, such as women from minority or indigenous groups, women with disabilities, women from lower caste groups or with lower economic status, migrant women. The Convention on the Elimination of All Forms of Discrimination against Women (CEDAW) requires that, ‘*State Parties shall take all appropriate measures to modify the social and cultural patterns of conduct of men and women, with a view to achieving the elimination of prejudices and customs and all other practices which are based on the idea of the inferiority or the superiority of either of the sexes or on stereotyped roles for men and women*’ [72, 73].

There is nothing to prevent health organizations from implementing CEDAW through their organizational governance and human resource functions. Strategies to eradicate stereotyping include: (a) using international, regional or national policy and rights frameworks as a basis for organizational governance and policy design; (b) providing human rights education; (c) naming and raising awareness of gender stereotypes, how they operate and their professional and social harms; and (d) strengthening individual and organizational capacity to challenge gender stereotyping in the design of human resource and organizational development strategies [74].

### Operationalize substantive equality principles in organizational governance and human resources management

Evidence of organizational gendering processes and the resulting systemic structural discrimination call into question the adequacy of gender-neutral organizational policies (were neutrality even possible). Gender neutrality sidesteps a fundamental truth, namely that insistence on gender neutrality by definition precludes protection for women ‘victimized by gender’ [59].

That is, gender neutral policy in overtly or covertly gendered work cultures that favor masculinist leadership and management stereotypes, male bias and a male ‘ideal worker’ norm, allows the uncontested and unimpeded operation of gender bias and discrimination, to women's disadvantage. Ultimately, a gender-neutral stance in gendered organizations permits the operation of processes and systems that offer *de facto* forms of affirmative action for the dominant and privileged group [75]. It should be noted that substantive equality involves preventing the application of gender-neutral laws in ways that have a discriminatory impact on women [76]. Promoting *substantive equality* policies and programs, such as target setting, quotas, affirmative mobilization and fairness, all mitigate this discriminatory impact [67]. (see Appendix 1, Key Definitions and Table 3).

#### Human resource policy and practice are gendered but hold promise for organizational change

As suggested earlier, organizations, organizational violence and discrimination and leadership are all gendered. Human resource practice is likewise gendered, but often unaware of it. Gender influences management interpretations of work commitment (where women are presumed to be less committed than men); the (de) valuation of flexibility (which affects job type and level, pay, working hours and contractual status); and the processes of selection, appraisal and reward [77]. Line management itself has been implicated as the ‘site of resistance to equality initiatives and an obstacle to women's career development’ [77]. Human resources management struggles to find effective approaches to backlash with respect to sexual harassment [30] and substantive equality measures like affirmative action. For example, the mixed results of ‘diversity management’ in eliminating discrimination has been subject to critique from many angles, including its failure to constructively manage backlash about, resistance to and hostility from both male managers and co-workers to equality initiatives such as flexible work-life programs [5, 78–80]. Some diversity management practitioners have tried to avoid backlash by ‘*degendering*’ the debate about flexible work. However, ‘degendering’ lessened the focus on, and the relationship between, unequal gender power relations and their consequences for historically disadvantaged groups in organizations [80]. In ‘degendering,’ it appears that human resource managers lost sight of why there was a lack of organizational diversity in the first place.

Although the evidence suggests that human resource practice is unaware of its non-gender neutrality, the practice nevertheless holds promise for organizational change. For this to happen, the gender and cultural blinders must come off, and the human resources management function re-imagined as a mechanism of social change. To realize women's human and labor rights in the course of achieving organizational goals, human resource policy and practice must first embrace principles of *substantive equality* (see definitions in Appendix 1). Second, human resource practice must undertake, in partnership with organizational leadership and employees, human- rights-based management strategies such as affirmative mobilization, affirmative fairness and special temporary measures (see Table 3). In practical terms, this also includes enabling conditions that result in greater ease in reconciling work and family life for all employees [27, 29, 81]. Third, the training of human resource managers must include developing skills in gender analysis, and orientation to new roles and knowledge of the special measures and enabling conditions needed to bring about substantive equality. To prepare effective organizational change agents, human resource training must produce practitioners who can explain and advocate for the principles, arguments and benefits of substantive equality to all organizational members, but especially to organizational governance actors; and to introduce change while anticipating and effectively managing backlash.

### Put in place special measures and enabling conditions to promote substantive equality and dismantle the mechanisms that contribute to women's gendered disadvantage in gendered organizations

Organizations that embrace substantive equality put in place special measures and enabling conditions such as those in Table 3 to protect their workers from systemic structural discrimination [67] and create egalitarian organizational cultures. Enabling conditions bring about egalitarian cultures by responding to the specific life-cycle needs of both working women and men, such as measures specifically for the support of maternity and paternity, which reduces gender segregation as it promotes work-life integration. Special measures (long or short-term) to prevent, end impunity and provide redress for sexual harassment, and close the wage gap [88] also promote egalitarian organizational cultures. Education about non- discrimination, sexual harassment, zero tolerance policies and codes of conduct, prevention and reporting systems, rigorous monitoring and enforcement to end impunity backed up by employer liability for policy infractions, and comparable worth policies to close the wage gaps associated with horizontal and vertical segregation [88].

#### Actions targeting work-family reconciliation

Enabling conditions that shift caregiving responsibilities in the family through work-family reconciliation policies are critical elements of organizational change for substantive equality. Examples of work-family reconciliation ‘good practice’ include paid family leave provisions, working time regulations, and early childhood education and care. However, not all flexible arrangements have the desired effects. For example, flexible work accommodations, usually individually negotiated arrangements between employee and manager, are prone to ‘flexibility stigma’ and small-scale effects [81, 85]. Work-family reconciliation should be designed with an understanding of the complex gender dynamics and rewards involved in ‘ideal worker’ norms, whereby male workers may be treated as both more capable and deserving of valued jobs and at the same time less capable of being caring parents [86]. Taking paternity leave violates the ideal worker norm and can put male employees who might opt for it at as much risk of being stigmatized as the female employees who, in leaving work early to work the ‘second shift’ at home, may be stigmatized as less productive and reliable. There are therefore built- in (organizational) cultural disincentives for both women and men to use these arrangements which must be addressed in communications and incentives for their use.

Because of the strength of gendered organizational rewards and sanctions, advocates recommend *deconstructing gender* [59] at work through long-term challenges to the ideal worker norm, work-family conflict and flexibility stigma. For example, in the National Science Foundation program described earlier [25], family-friendly policies, dual-hire programs (rather than ‘trailing spouse’ accommodations), making resources available for child-care, automatic clock stoppage for *all* faculty with the birth or adoption of a child or other qualifying event, were considered as primary ways to shift organizational expectations of a standard career path for the male ‘ideal (research) worker.’ Mentoring and networking that targeted individual professional development were considered (only) secondarily.

Deconstructing gendered work also includes collective work redesign models that alter the structure of work, working groups and organizational culture [25, 27, 29, 56, 59, 81]. Examples include Predictable Time Off (which was not connected to HR) and Results-Only Work Environment, which asked departmental work teams to critically reflect on the traditional model of work and identify new effective ways of working together that focused on outcomes [29]. Collective efforts to integrate work and family can overcome the flexibility stigma attached to individual accommodations and the powerlessness experienced by many of women at work [93].

#### Action targeting research

Not surprisingly, the gender biases in organizations find their way into research content and processes [63]. The following are examples of gender biases in research: Not collecting sex-disaggregated data; the use of gender-blind methodologies; women's under-representation in clinical trials and in leadership of research communities, ethical committees and advisory bodies; and the differential treatment and funding of woman-led research [63]. The processes of gender segregation and stereotyping that push women out of science, technology, engineering and mathematics careers [41] also contribute to a risk that what is produced as health knowledge is as gendered as the organizations that sponsor it. Gender analysis with a concern for intersectional inequalities is a first step in taking off some of the cultural blinders.

#### Actions targeting leadership

To understand what makes a leader effective in an organization, the gender of the leader and the conduciveness of the organization to women's leadership must be taken into account in gender analysis [32]. The ways that leadership is defined to favor masculine-typed traits creates barriers for would-be women leaders. Organizational governance leaders committed to promoting women's representation in senior roles should consider both the current type of leadership model and alternative ways to organize work to enable leadership and self-management to be enacted more broadly and at different levels of organizational life.

#### Leadership models

If the model of organizational leadership continues to reflect stereotypes of the powerful ‘agentic’ male, then women who want to ascend to senior levels will likely continue to face the double binds and backlash mentioned earlier. However, if an organization adopts transformational leadership models that de-emphasize the command and control strategies traditionally associated with ‘agentic’ male leadership [32], then there may be more opportunities for women to assume leadership roles. However, if gender-neutral policies function as ‘*de facto* affirmative action for dominant groups’ [75] in gendered organizations, then the utility of a gender-neutral leadership model, albeit ‘transformative,’ should be reconsidered. For example, a model of feminist leadership [83] the protection of women's labor/employment rights and the promotion of social justice, might be effective in addressing women's gendered disadvantage within the organizations. In any case, changing the organizational model(s) of leadership is more important in initial stages than (even relevant) micro-level interventions such as leadership training and mentoring.

#### Alternative ways to organize work

There are also alternative ways to organize work that do not depend on the model of a powerful ‘agentic’ male on top of a traditional hierarchy, controlling and sometime coercing the workers and work products of the organization. This traditionally hierarchical way of working undermines self-management and the taking of leadership initiative at lower levels. To disrupt this pattern, an organization (i.e., its governance an management functions) would restructure the ways it organizes work, shifting away from *hierarchy* (or pure hierarchy), where there is a leader who exerts control on organizational directions or work products (such as research studies), to non-hierarchical models of organizing work such as the dispersed ‘rule’ of *heterarchy,* or the self-governance of *responsibly autonomous* teams [84]. Examples of *heterarchical* practices include rotating director positions every few years, making major strategy decisions with representatives from sub-units, or having an organizational governing council made up of representatives of sub-units. Responsible autonomy is self-government or self-organization in the absence of external control of work, though there is accountability for outcomes. This way of organizing work seems particularly congenial for academics and health researchers [84].

## Changing gendered organizations: taking stock, moving forward

Global health organizations, like all organizations, are inequality regimes characterized by systemic, structural obstacles for women striving to assume senior leadership and participate in more varied roles in organizational life. Leadership, violence, work design, human resources management and research are gendered experiences in organizations. The type and amount of ‘career capital’ women can accumulate are likewise gendered. Future global health research and gender analysis should examine the structures of gender inequality regimes in health organizations, including the co-occurring gender distinctions, biases, exclusions and relational patterns that are barriers to representation at senior levels. Change efforts will involve testing nuanced and deliberately transformative organizational strategies to dismantle embedded, discriminatory processes and structures of privilege and unequal opportunity. It is likely that multiple strategies such as presented in Tables 2 and 3 are needed to equalize access and opportunity for leadership and more varied roles, which will require resources and support from organizational governance and management structures. Strategies should primarily target the organizational level, aim to establish substantive equality policies and programs, and create enabling conditions that ‘deconstruct gender,’ especially the male ideal worker norm and other hierarchical patterns of work. Table 4 summarizes organizational development strategies to bring about more accountable gender -egalitarian global health organizations.

**Table 4.** Actions to bring about more gender egalitarian global health organizations

Visioning and enacting the organization based on egalitarianism principles and commitment to maximizing members’ competencies and engagement. Participatory gender analysis for organizational learning related to systems, culture, leadership and power. Organizational restructuring and work redesign including special measures and enabling conditions to bring about substantive equality. Organizational culture free of harmful gender stereotypes and coercive power. Woman- and family-friendly human resources policy and practice. Transformative leadership models and feminist leadership development. Alternative, non-hierarchical ways of organizing work that allows self- management and leadership initiative on the part of all organizational members. Indicators to monitor accountability to women's human and labor rights

Efforts to realize gender equality have met with resistance, a lack of political will, political opposition [94, 95] backlash [96, 97] and ‘policy evaporation in the patriarchal cooking pot’ [97], which operate in organizations as they do in the larger society. Indeed, advocates who have reflected on the successes and failures of gender mainstreaming [and stalled or intermittent progress in women's rights] point to denial that there is a problem of women's subordination, pervasive gender discrimination that is unfavorable to women but unwillingness to take action on it, and a lack of commitment and accountability, which raise an inescapable question: At bottom, is there true acceptance of the equal worth of women and men [98]? This question should be revisited periodically in efforts to redress the gender imbalances in senior global health leadership roles.

Moving forward, there must be a vision of egalitarianism and equal rights in global health organizations that puts women at the center of organizational evolution towards substantive equality. Women must initiate, actively lead, advocate and negotiate over the long term for needed changes [76] from wherever they are placed, and with whatever power they have, in their organizations. As one social justice activist put it, freedom is a constant struggle [91]. And if the barriers to substantive equality are political, then political objectives and strategies are needed.

Dismantling the ‘legal apparatus’ of gender segregation [91] will require laws and policies against discrimination and violence that can become transformational organizational policy. However, the transformational power of policy will ultimately depend on advocacy, organizing, collaboration and synergies inside the organization, between organizations, and with autonomous, anti-authoritarian feminist coalitions and movements in the larger community [90]. As with other types of segregation and inequality [91], eradicating gender segregation and inequality will require longer term efforts targeting culture change (inside and outside the organization) that are inclusive, and that identify commonalities in diversity, in order to diversify the bases of solidarity. Patriarchal structures and attitudes begin to lose their legitimacy and normalcy only over time (and generations) as new forms emerge to challenge them [69]. With each challenge, over time, one paradigm replaces another. The paradigm can shift when everyone participates in ways that make unequal systems happen differently, in ways that tip the scales towards new paradigms [69] of equality.

Two guiding questions should be addressed in organizing and strategy development efforts: What are appropriate forms of resistance to the subordination and marginalization of women that do not reinforce paternalistic and patriarchal logics in organizations? And, who will be effective allies in formulating and enacting these new forms of resistance [92]? Backlash should be anticipated and can be managed by integrating risk assessment and mitigation at the front-end of strategies and monitoring for adverse outcomes [99]. However, resistance need not be confrontational. For example, the Women in Global Health *60/40 Gender Parity Panel Pledge* [100] links organizational advocacy to global advocacy in what is essentially an effort to *desegregate* global health leadership in international professional communities.

## Conclusion

Organizational inequality generating mechanisms are driven by cultural norms that, to one extent or another, subject women to subordination, discrimination and violence in organizations. To greater or lesser degrees, organizations feature systemic structural discrimination that shapes privilege and status and make women's opportunities, conditions and experiences substantially different from men's, and substantially disadvantaged in comparison. All these have implications for the extent to which women can participate in varied roles and senior representation in global health organizations. All these undermine women's chances to play leadership roles in the achievement of organizational and health development goals.

Undertaking organizational change may seem a utopian dream. But people can bring into their organizations any positive evolution towards egalitarian relationships and substantive equality that exist in the larger society, just as they bring the gender inequality order of the larger society into their organizations. Ensuring ‘*women's full and effective participation and equal opportunities for leadership at all levels of decision-making in political, economic and public life*’ [1] can begin in the global health organization. Interested organizational actors and their allies can advocate for, initiate and lead changes in the global health organizations in which they work, starting with any of the policy and practice changes proposed here.

## Figures and Tables

**Figure A1.**
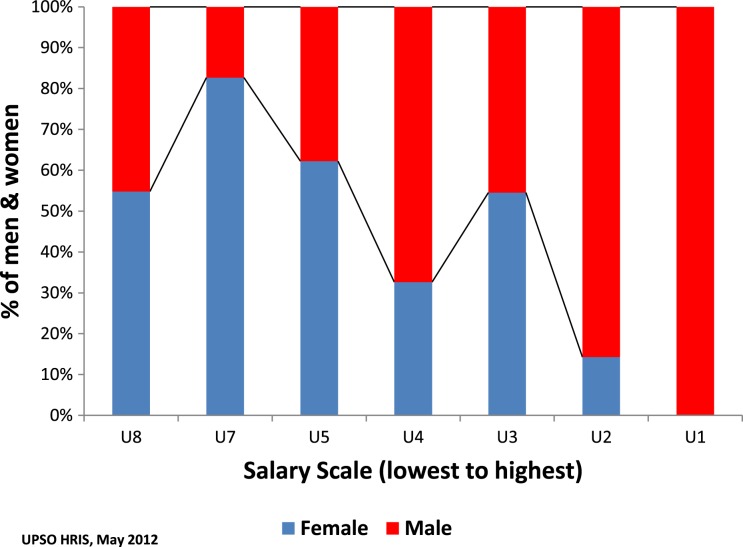
Percentage of Men and Women by Position and Pay Grade in UPSO Regional Referral Hospital (*n*=183)

## Appendix 1

**Table:** Key Definitions^a^

***Discrimination***: Any distinction, exclusion or restriction made on the basis of sex which has the effect or purpose of impairing or nullifying the recognition, enjoyment or exercise by women, irrespective of their marital status, on a basis of equality of men and women, of human rights and fundamental freedoms in the political, economic, social, cultural, civil or any other field.
***Enabling conditions***: Institutional arrangements that seek to redress the fact that embedded preferences for privileged groups are already built into institutions. Enable structurally disadvantaged groups to access opportunity, which supports the achievement of *substantive equality.* Include services (e.g. child care); structural policies (e.g. maternity/paternity leave); and institutional remedies to overcome and deter discrimination.
***Family responsibilities discrimination***: Exclusions, restrictions, or distinctions against individuals (such as pregnant women, mothers and fathers of young children, parents of disabled children, and individuals who care for their aging parents or sick spouses/partners) based on their responsibilities to care for family members.
***F**ormal equality***: Promotes gender neutral policies and equal treatment based on merit (which is gendered) for groups that are supposed to be of equal status but that are not because of historic discrimination. Equal rules for unequal groups can have unequal results.
***Gender hegemony:*** Culturally dominant beliefs and norms related to the ideal relations between men and women, masculinity and femininity.
***Health organization:*** The formally planned, coordinated and purposeful action of human beings to achieve a mission, goals or objectives in the area of health and wellbeing. The organization—from multi-lateral, to academic to governmental to non-governmental--features hierarchies of power characterized by hegemonic masculinity intersected by other axes of social inequality that are salient in that culture (e.g., race, ethnicity, class, religion, age, etc.).
***Hegemonic masculinity:*** The masculine position that is dominant among multiple configurations of masculinity that are hierarchically organized along lines of domination (of men over women, of powerful men over less powerful men, of adult men over younger men). Generally associated with the subordination and oppression of women. A form (or forms) of “emphasized femininity” has been postulated, characterized by women's accommodating the interests and desires of men (i.e., compliance with the unequal structuring of gender relations and collusion in the unequal distribution of power). Other forms of masculinity and femininity may exist, shaped around strategies of resistance or cooperation. In a male dominant gender order or organizational regime, masculinity is defined through a difference with femininity, and femininity is a position of subordination in relation to masculinity. The specific features of masculinity and femininity that ensure men's dominance over women as a group varies depending on context and must be analyzed in each cultural setting.
***Intersectionality:*** A feminist theory and analytical tool for understanding and responding to the ways in which gender intersects with other identities to create new oppressions. The experiences of marginalization and privilege are not only defined by gender but by other identity factors, such as race, class, age, religion and sexual orientation to name a few—all of which are determined, shaped by, and imbedded in social systems of power. Intersectional paradigms view race, class, etc. as mutually constructing systems of power that require special measure to reach women who face multiple forms of discrimination.
***Pregnancy discrimination***: Exclusions, restrictions, or distinctions made on the basis of pregnancy, childbirth, or related conditions, such as unwillingness to hire, promote, or retain female students or workers who may get pregnant and leave school or the workforce or who require maternity leave and benefits. Pregnancy and family responsibilities discrimination are related forms that target a broad range of reproductive functions and circumstances that may be viewed together as ***reproductive role discrimination***. These related forms of discrimination usually target women of childbearing age who are not able to equally access opportunities for education, hiring, or promotion.
***Sexism:*** The ideology of male supremacy (or superiority) and the beliefs that sustain it. Patriarchy is sustained by sexism.
***Systemic structural discrimination***: Patterns of behavior, policies or practices, and social, economic or cultural background conditions that are part of the structures of institutions, which create or perpetuate disadvantage for members of a marginalized group relative to other groups in society or organization. Created historically through past discrimination, entrenched in institutions, it includes the gendered division of labor and gendered violence. The most persistent obstacle to the achievement of substantive equality and therefore the primary focus of temporary special measures. Effective temporary special measures should aim at ending structural discrimination.
***Special temporary measures***: Programs, policies and laws that seek to neutralize and redress embedded structures of discrimination and preferences for privileged groups that are already built into social institutions. Such affirmative measures place women or other marginalized groups in a situation of comparative advantage for a limited period, with the aim of achieving substantive equality in the long term.
***Stereotype:*** A generalised view or preconception about attributes or characteristics that are or ought to be possessed by, or the roles that are or should be performed by, members of a particular social group. Affects employment decisions and career progress.
***Substantive equality***: A principle that takes into account the effects of past discrimination, recognizes that rights, entitlements, opportunities and access are not equally distributed throughout society, and the need to sometimes treat people differently to achieve equal results. Achieved by preventing *systemic discrimination* by adjusting policies and practices to meet the specific needs of certain groups. Recognizes that the neutral, gender-blind character of formal equality masks structural discrimination and privilege that are embedded or built into institutions as a result of past discrimination. Allows for differential treatment to level the playing field for women, particularly where structures of dominance and subordination are embedded in the baseline of opportunity. Differs from formal equality by entitling women to equitable outcomes and access to social goods (e.g., education) on an equal basis with men (i.e., equality of results as well as opportunity).

a See References: UN General Assembly, 1979 & 2012; Crenshaw, K, 1993 &1999; Cusack, 2013; and International Women's Rights Action Watch Asia Pacific, 2006. Also see: https://en.wikibooks.org/wiki/Introduction_toSociology/Organizations. For hegemonic masculinity and femininities, see Schippers, M., 2007 and Jewkes, R & Morrell R, 2010, For sexism, see Lerner, 1980.

## Appendix 2

**Appendix 2.1. Background**

**Appendix 2.2. Analysis of personnel data**

**Appendix 2.3. Focus Group Data**

**Appendix 2.4. Organizational Action Plans**

### Appendix 2.1. Background

Supplementary materials consisting of unpublished excerpts from gender analyses in two African health institutions shed light on the *non-gender neutrality of organizational structures and culture*, including gender segregation, gender stereotyping and overt pro-male bias in recruitment, hiring, and promotion, the denigration of pregnancy and family responsibilities, the intrusion of socio-cultural patterns in organizational HR hiring practices with consequences for unequal employment opportunity, sexual harassment and the stigmatization of affirmative action.

Appendix 2 materials maintain the requested anonymity of the health organizations and by following examples from other peer-reviewed journals^2^ are referred to as Zambia Private Sector Organization (ZPSO) and Uganda Public Sector Organization (UPSO),

Between 2011 and 2014, UPSO and ZPSO analyzed and improved their efforts to advance gender equality in their programs, administrative functions and institutional cultures, by employing a multi-method approach to organizational gender analysis. The materials in this appendix are based on a subset of the gender analysis data generated by the *analysis of personnel data* related to equal opportunity and the concentration of men and women in different types or levels of jobs and occupations, and by *focus group discussions* to obtain a wide range of perceptions, and experiences of male and female staff related to equal opportunity, gender equality, organizational policies, sexual harassment, and affirmative action.

*Data analysis:* Content analysis of the focus group data employed Nvivo to identify key themes and subthemes. Researchers coded the focus group data by breaking down transcripts into quotes or text units, sorting according to thematic categories, assessing the validity of coding, and resolving inconsistencies. The analysis of data was guided by the Gioia method,^3^ including 1) generating “first order concepts” (i.e., data expressing informants’ terms and understandings) 2) identifying “second order themes” (i.e., abstract level themes and a larger narrative describing “what is going on here?” in theoretical terms), and 3) identifying larger dimensions that might help explain various themes suggested in the data. After coding, the themes, concepts, dimensions and their interrelationships were organized iteratively. Cross tabulation of the personnel data used SPSS.

*Ethics:* Ethical reviews determined that the gender analysis, conducted for organizational learning and program improvement, had minimal risk to human subjects and was thus exempt from further ethical review. The gender analysis tools were vetted by the two health organizations and revised based on leadership and staff feedback.

### Appendix 2.2. Analysis of personnel data

Appendix 2.2 features selected results from the analysis of personnel records from ZPSO and UPSO to explore patterns of occupational segregation. This analysis revealed the vertical occupational segregation associated with “the glass ceiling.”

**ZPSO:** The employee *population* was fairly well-balanced overall, with female employees comprising just under half (46%) of the workforce, and males 54%. One might expect roughly equal proportions of men and women at different position levels in the organization *if there were no occupational segregation.* However, at the time of the analysis, the patterns depicted in Table A1 point to segregation by position level (B, D, F, and H) and vertical segregation in senior representation (Level B).

**Table A1.** Concentration of men and women by position level, ZPSO, 2012 (N=364) (Job category A is not applicable because there is only one person in the job).

Position level*	Male	Female	Pattern
B	**75%**	25%	Segregated
C	54%	46%	Integrated
D	**68%**	32%	Segregated
E	47%	53%	Integrated
F	**71%**	29%	Segregated
G	42%	58%	Integrated
H	**70%**	30%	Segregated
I	54%	46%	Integrated

**UPSO**: The health employees were a subset of the larger population of public service sector employees. Women comprised 57% of the health employee sample, and men, 43%, indicating a preponderance of female health workers. If there were no occupational segregation in the public health sector, one would expect to find roughly the same gender proportions of men and women reflected in each job level and category shown. Instead, vertical segregation was evident in the percentages of men and women occupying various hierarchical levels depicted in Table A2, with male employees concentrated in senior and middle management (U1-U3) and especially at the top (U1) (77% male). There were more women concentrated in the lower and middle levels (61% in U4–5 and 57% in U6-U8).

**Table A2.** Number and percentage of women and men concentrated in public sector health workforce jobs in eight districts and four national-level facilities, UPSO HRIS, 2012 (N=6,450)

Salary scale	Number of employees	Male	Female
Senior management level (U1)	133	77%	23%
Middle management level (U2-U3)	326	63%	37%
Graduate and diploma entry level (U4-U5)	2,406	39%	61%
Lower level (U6-U8)	3,585	43%	57%
**Total**	**6,450**	**43%**	**57%**

Similar patterns of vertical segregation were found in UPSO regional hospital sites, as illustrated in Figure A1, where men occupied positions in the highest pay grades.

In summary, the analysis of the personnel data substantiated vertical occupational gender segregation. Focus group results in Appendix 2.3 illustrate the gender beliefs and stereotypes that underpin the apparent trends in vertical segregation.

### Appendix 2.3. Focus Group Data

This appendix features selected results from the ZPSO and UPSO focus groups discussions (FGDs) to illustrate the ways that gender stereotyping serves to create or maintain gender inequalities such as vertical segregation that is biased towards men's leadership, to rationalize the marginalization or exclusion of female employees based on pregnancy and family responsibilities, and sexual harassment that subordinates female targets. The focus group data also demonstrate that organizational processes such as hiring and promotion, and interpersonal relations, are in no way “gender-neutral” and that there are both implicit and explicit gendering processes and mechanisms that shape opportunity, privilege, (subordinate) status and treatment. ZPSO FGD data are supplemented by UPSO FGD data.

Major themes included:

1. The intrusion of socio-cultural patterns, including gender norms and expectations related to women's role as wife and mother in the gender division of labor, into organizational HR hiring practices, with consequences for unequal employment opportunity (e.g., such as when women were not offered positions by hiring managers in anticipation ‘that husbands will refuse.’).
2. Denigration^4^ of pregnancy and family responsibilities which marginalizes pregnant women and working mothers, also disqualifies them women for leadership or desired jobs, and contributes to a glass ceiling.
3. Gender stereotyping that serves to contrast male and female workers’ productivity, reliability, temperament and competence, and makes claims about men's greater suitability and availability for leadership and other valued jobs
4. Overt (pro-male) bias in recruitment, hiring, and promotion processes, linked to the “ideal worker” norm.
5. The stigmatization of affirmative action seems to pit unqualified beneficiaries (i.e., women) against competent employees (men) who, in their management roles, act as gatekeepers of organizational quality standards.
6. Quid pro quo sexual harassment as an abuse of power and control of female subordinates which results in feelings of vulnerability and ‘suffering in silence,’ with stereotypes of men as unable to control themselves in the face of women's sexual provocation.

#### Illustration of Themes, Narrative and Discussion

Intrusion of socio-cultural patterns, including gender norms and expectations: Employees in the **ZPSO** focus groups indicated that husbands’ traditional expectation of wives shape women's work options, that they use their prerogative as head of household to approve or veto employment and advancement opportunities. As one **ZPSO** female participant stated, “*There are few women* [*who*] *can go just independent.”* Hiring managers made decisions based on these cultural patterns, as another woman explained: “Women may not be offered positions, such as area managers, or outside Lusaka, in anticipation by managers that husbands will refuse.” A woman manager observed that women especially lose out on “top” opportunities:
Those top jobs, you might be asked to work from another town and probably your husband won't allow you to go out of town. Therefore we are denied, we face a challenge…”

Denigration and marginalization of pregnant women and working mothers in the workplace: ZPSO FGD participants made numerous comments that illustrated the ways in which women workers of reproductive age are denigrated, marginalized and disadvantaged in the organization. A female **ZPSO** manager explained the absence of women in higher management this way: “They think that if we give this job to her, she may have babies the next day, maternity leave, breastfeeding, [and] what are we going to do when she is not around?” A male employee from **UPSO** suggested that “mood **swings, periods,** [and] **maternity**” negatively affects women's work, and that **men** are **more “versatile**.” Another **UPSO** male employee remarked, “Women have…issues like pregnancy and…maternity leave” and “therefore…would not be suitable for …a high position that requires a lot of responsibility.”

Perceptions of women's lower productivity and reliability contrasted with perceptions that men are better able to “reach targets” and “accomplish goals” than women who have competing domestic responsibilities at ZPSO. Several male employees unfavorably compared female versus male attendance, suggesting that “men…will be here [but] women will just go [home].” Another male employee quantified male attendance at 90% and added, “For women, I would give it 45%.” A male manager also admitted that because women concern themselves with problems at home, men don't have to:
As a woman, your mind can be obscured by problems which you left at home, children who are not feeling well, every half an hour you have to call home, you stop working, make a phone call, find out, but for us men, over half of the time, we expect women to be doing that. While we avail ourselves to work.

Pro-male bias in recruitment, hiring, and promotion: The tendency to exclude women from consideration for outreach positions was reflected in numerous comments across focus groups. A **ZPSO** male employee candidly remarked about field work:
If you look at the nature of the work there, it involves being on the road for days. So when you look at an average woman and going back to our culture, …like even when it comes to applying, they might get a bit of resistance here and there. I would say…it might be natural discrimination.

When asked “If there were two persons, a man and a woman, equally qualified for the position of ‘director,’ who would be more likely to be given the job by the recruiting authority?,” male **UPSO** employees and managers agreed that men would be more likely chosen, explaining that men were more *inherently fit for leadership*. A **ZPSO** female manager noted “…They feel a woman can't manage, it is going to be a tough job, often out in the field, so we prefer to get a male candidate.” Regarding advancement, **ZPSO** female focus group participants also suggested that promotion “has nothing to do with qualification [or leadership qualities]; it has mostly to do with gender.” A female manager observed:
*I haven't seen any female being promoted to a higher level. It has always been male…. …A manager who resigned…had his own recommendations for a female candidate. He presented it to management, but management said, no she was female, and they had to get somebody from outside*.

A female **ZPSO** employee remarked that the organization considered family responsibilities when deciding whether to renew women's contracts: “If you miss work numerous times, maybe you are sick, or your child is sick, they consider all those things.”

Gender beliefs and stereotyping that make claims about men's greater suitability for leadership and most jobs: Male focus group participants from **ZPSO** used the words “natural” or “naturally” repeatedly, even in connection with discrimination, suggesting that “**By nature, men are born with leadership quality.**” A male manager put it this way:
If two candidates are equally qualified, naturally it is wiser to give the position to a man……. A man is **more…mentally agile** than a female.

Gender beliefs and stereotypes that negatively characterize women's productivity, reliability and leadership competence: Unequal opportunity for advancement into management was linked to feminine stereotypes, prejudices against women, and resistance to women's leadership. As one female employee from **UPSO** commented “…The process of uplifting women is still ongoing, and there are still some doubts about women's performance at [the] leadership level.” Female managers in **ZPSO** agreed that gender beliefs operate even in the presence of lip service to equal opportunity, with negative employment prospects. For example,
*If an ad was put today, and we write the usual, “We are an EOE* [*equal opportunity employer*]*,” and women apply, when it is time to shortlist, …if the head of that requesting department is already putting up all these hurdles, saying “Women do this,” “They are gonna take maternity leave,” “She is gonna get pregnant,” she might have applied…but there is a ceiling for her.*

Male ideal worker norm: This norm was invoked to rationalize preferential hiring of men into valued jobs. For example, some positions in the **ZPSO** (such as area manager or outreach worker) called for long-distance travel and long working hours. Focus group participants suggested that men were more able to perform as ideal workers in this regard. For example, a ZPSO female employee remarked,
*…Males…are **more trusted**.* [*…*] *Males will still come* [*to work*] *even when the baby is sick. Males don't breastfeed, they don't bathe the baby, they don't wash nappies. They are treated differently.*

Some **ZPSO** participants shared perceptions about women's unsuitability for leadership positions which were attributed to women's “letting personal feelings come into their work,” though female participants tended to contest the unfairness of the emotionality label. One female **ZPSO** manager cited a double standard, saying, “You'd be raising a point, and when you are in a situation where there are more men, because it is contrary to what they are saying, they say ‘Women are emotional,’ and try to bring it down to that level.” Another **ZPSO** female manager suggested that women's expressions of emotion in the workplace do not preclude good decision making:
…Women are…looked at as **emotional beings**, maybe because we express our emotions easily, we don't hide…. …But that doesn't affect our decisions, just because you are emotional, you are not going to make wrong decisions. …I can be pissed off, like a male manager can be … some people are going to say, “It is because she is a woman.” But most probably if it was a guy in my situation, he was going to do similar things and nobody was going to say, “It is because he is a man.”

Tables A3 and A4 present language used by men and women in both organizations to illustrate essentialist stereotypes of male and female health workers and leaders.

**Table A3.** Gender essentialist and male primacy stereotypes about male and female workers and leaders : ZPSO

ZPSO Male stereotypes	ZPSO Female stereotypes
“By nature, men are born with leadership quality.” “A man is more mentally agile than a female” “More confident” “More trusted” “More versatile” “More productive” “Better decision-makers” “Men have a biological makeup that makes them vulnerable to appearances”, “Men by nature easily get moved when they see certain things”…”goats they have to feed on grass.”	“Helpers” “More unorganized [and] more irresponsible” “Emotional beings,” “Rule using emotions and not what is on the ground” “Can't manage” “Not really qualified” “Female managers, shut you down so you can't argue with them” “Seek revenge towards male staff” “If we give this job to her, she may have babies the next day” “As a woman, your mind can be obscured by problems you left at home” “Women are really suggestively dressed”

**Table A4.** Gender essentialist and male primacy stereotypes about male and female workers and leaders: UPSO

UPSO Male stereotypes	UPSO Female stereotypes
“Faster in thinking” “Logical” “Detached” “Have more wisdom” “When men are bosses, they think they can take anything from female subordinates, so they start asking for sexual favors”	“Make more mistakes” “Mood swings” “Not competent enough” “Women have issues like pregnancy …and therefore would not be suitable for a high position that requires a lot of responsibility”

Male resistance to women's leadership was also in evidence in some **ZPSO** FGD participants’ generalizations about female behavior in management jobs. For example, “The female managers **shut you down** so you can't argue with them. …With the male managers, you can argue a bit.” In addition, women department heads are seen to be compelled to “show power” and “prove themselves”, and one male employee implied that women should be grateful for the “favor” of leadership:
*…When you put* [*women*] *in managerial positions, they try…to seek revenge towards male staff. …If you have got an issue and you raise it to your female managers, the first thing* [*they*] *will do is not to look at the successes, but they will start with the shortfalls. You try to answer, she increases her voice. …We are trying to do a favor to you because all along you have been stamped on your toes. Now that we want to elevate you to this level, can you not bring in attitude?*

In the face of this, most female managers appeared to believe that they needed to prove themselves and be “firm” and “tough” so as to “control the situation.” As one female **ZPSO** participant observed, “Sometimes people take advantage, especially when she is a woman. They will say, ‘She is soft, she will give me the permission.’ Sometimes, she should be a bit tougher.”

Stigmatizing affirmative action as assistance only needed by the unqualified : In **ZPSO** focus groups, there appeared to be an underlying assumption that affirmative action opens the door to unqualified (female) candidates. A female employee commented, “I don't think it is right for a woman who is **not really qualified** to be at a position where she is not supposed to be, just because she is woman.” A male manager similarly stated,
…If it means being equal, let's compete. It shouldn't come just because you are a female. It should come because you are able to handle it, you are qualified, and you are up to the task…that's what I consider gender equality.”

Another **ZPSO** male manager evoked a perceived conflict between promoting equal opportunity and doing what is best for the organization:
…We find an application letter, I would want to maybe to encourage women to be called, but then I also look at their qualification, and I don't want to use that as a process of disadvantaging the organization.

Sexual harassment as gendered power and subordination: A narrative of blaming women for the occurrence of sexual harassment and responsibility-shifting was discernible from the **ZPSO** data. ZPSO discussions of sexual harassment highlighted a stereotype by men about men, that they are unable to control themselves in the presence of women who are perceived as “provocatively” dressed. For example, a **ZPSO** male manager stated:
*…**Men have a biological make-up that makes them vulnerable to appearance**. So we can ignore … but I think there are some cases where **women are really suggestively dressed**, and it is difficult because it creates an environment which is very hard…because men mostly, we go for what we see*.

ZPSO male FGD participants advocated for a stricter dress code to deal with women's provocativeness. One male manager put it this way:
We are admitting a weakness, but…women can help us not to jump the gun, because there is saying in our African culture, you know **goats they have to feed on grass,** now you know this is your lawn, then you take your goat and tie it there, and your grass will be eaten up. Who has caused that? It is you, the person, so women must understand that men by nature easily get moved when they see certain things. So women can help us a great deal by just being modest. It is not that we can't control, yes we can, but it will be an additional help.

**UPSO** focus group participants’ comments suggested that sexual harassment is recognized at least by some as a mechanism involving gendered power and subordination. A **UPSO** female employee stated, “*When men are bosses, they think they can take anything they want from female subordinates,* so they start asking for sexual favors.” A **UPSO** male manager pointed out that “some managers ask for sex in order for one to be promoted,” In **UPSO** focus groups, participants said of victims’ responses, “Some decide to ignore it while others suffer quietly” while other “…people quit their jobs.” While some of the **UPSO** FGD participants denied sexual harassment occurred, one male employee noted that “Sexual harassment is silent; no one discloses.” A female manager remarked:
*I do not agree that sexual harassment is not common. I was a victim. But I failed to* [*find out*] *where to report* [*it*]. *Is there a way we could find where to report? I don't see it in any policies*.

A **ZPSO** male employee observed that a woman who “stands her ground” runs the risk of a bad evaluation or job loss. Sexual harassment thus presents as a gendered obstacle to career advancement and the accumulation of social and work capital.

**Discussion**: The FGDs identified frequent examples of gender essentialist and male primacy stereotyping in the creation and maintenance of gender inequality in these two organizations. They demonstrated *“claims-making*” relevant to the creation of status hierarchies such as the glass ceiling where, on the presumption of greater competence, men claimed greater organizational authority, respect, resources and rewards and thus, dominance in organizational culture through formal leadership.

The FGD narratives also suggest constellations of negative stereotypes about female co-workers and senior managers, manifest in references to women's emotionality, mood swings, tendency to make mistakes, lesser productivity and commitment, unreliability, vengefulness, lesser mental agility, inability to handle power, and incompetence. The link between pregnancy and the glass ceiling, where having babies (or having the capacity to have them) seems to portray women as unqualified for promotion. This finding suggests the importance of pregnancy in hiring and promotion, and not just in firing, decisions. The linked processes of negative stereotyping of women and marginalization based on reproductive roles perpetuates inequality of opportunity to participate in paid work itself, not to mention senior level representation and leadership.

The focus group excerpts from ZPSO suggest victim-blaming in sexual harassment even as cultural and organizational norms target women with non-professional, sexualizing and subordinating behaviors, based on assumptions of female sexual provocation and acquiescence, unregulated by organizational accountability systems (e.g., policy, reporting systems, etc.), which made women's work conditions and experiences substantially different and disadvantaged based on their sex.

Finally, affirmative action was discussed in the ZPSO focus groups as if it might disadvantage the organization by pitting unqualified beneficiaries (i.e., women) against competent employees (men) who, in their management roles, must act as gatekeepers of organizational quality standards. If affirmative action is framed as unfairly favoring undeserving or incompetent candidates, it distances managers and employees from its legitimate uses, and leaves gendered privilege uncontested and firmly in place.

### Appendix 2.4. Organizational Action Plans

#### ZPSO's gender equality action plan

After the results were disseminated, ZPSO responded to drivers of inequality by developing an equal opportunity and gender equality policy which recognized substantive equality, and an implementation plan that targeted the specific inequalities highlighted by the gender analysis. This policy endorsed affirmative action, promoted family-friendliness to respond to employees’ lifecycle needs, prohibited discrimination based on pregnancy and family responsibilities, and outlined organizational processes and accountabilities to prevent and respond which to sexual harassment. By the end of the analysis and the ensuing internal dialogue, ZPSO had:

- Increased female employee presence in senior positions and male-dominated departments (e.g. motor pool, regional managers and research and levels (senior management) and male-typed jobs.
- Coordinated employment actions with the national Labor Office to increase accountability.
- Modified volunteer recruitment using clearly- defined criteria to increase female participation.

#### UPSO's gender equality action plan

UPSO convened a multisectoral Human Resources Technical Working Group to review and debate findings and interpretations and disseminated results at a national meeting for multiple stakeholders. UPSP subsequently convened a multisectoral equal opportunity and gender equality task force to plan for sector-specific, structural responses, one of which was to develop and disseminate a set of *Guidelines for Mainstreaming Gender in Human Resources Management*, which promotes equal opportunity, affirmative action, human rights, substantive equality and family-friendliness in health sector workplaces, with indicators for monitoring at central and decentralized levels. These guidelines were subsequently disseminated at decentralized levels and integrated in the annual work planning of district-level health managers. A sexual harassment formative assessment was conducted and information is being used to design a pilot sexual harassment prevention and response system.

## Appendix 3: Analysis of Gender and Intersecting Inequalities

This appendix provides more information on relevant articles with guidance on how to understand and analyze intersecting inequalities in health research. It also offers thoughts on an organizational gender analysis approach that addresses intersectional oppressions.

### I. Relevant articles on analysis of intersecting inequalities

The field of intersectionality analysis faces theoretical contradictions and ambiguities, methodological and practical challenges (including ongoing debates about how to define and measure the categories that “intersect”).^5^ As it is described in current scholarship, intersectionality appears to have been applied to the analysis of health inequities through health research, focusing on health experiences, conditions and access to services, not organizations. The following resources highlight issues and debates:

1. Springer, KW, Hankivsky, O and Bates, LM. Gender and health: Relational, intersectional and biosocial approaches. Social Science and Medicine. 2012. 74 (2012) 1661–1666.
2. Tolhurst, R. et al. Intersectionality and gender mainstreaming in international health: Using a feminist participatory action research process to analyse voices and debates from the global south and north. Social Science & Medicine 74 (2012) 1825–1832
3. From intersectionality to interference: Femnist onto-epistemological reflections on the politics of representation. Geerts, e and van der Tuin, I. Women's Studies International Forum. 41 (2013) 171–8.
4. Iyer A, Sen G, and George A. The dynamics of gender and class in access to health care: Evidence from Rural Karnataka, India. International Journal of Health Services. 2007. Vol 37, No 3. 537–554
5. Caiola, C, Docherty, SL., Relf, M, and Barroso, J. Using an intersectional approach to study the impact of social determinants of health for African American mothers living with HIV. 2014. Advances in Nursing Science, Vol. 37, No. 4, pp. 287–298
6. Hankivsky, O. Intersectionality 101. 2014. Institute for Intersectionality Research and Policy.
7. Hankivsky, O. (Ed.). Health inequities in Canada: Intersectional frameworks and practices. Vancouver: University of British Columbia Press. 2011.
8. OXFAM Intersectionality Practice Papers Series (with four (4) papers):
  - Maseko, S and Ndlovu, S. “Your struggle is my struggle”: Integrating intersectionality in work with lesbian women, bisexual women and trans-women in Zimbabwe. 2015.
  - Enarsson, J. R. Fe-politicizing intersectionality: How an intersectional perspective can help ONGOs be better allies to women's rights movements. 2015
  - Ruiz D and Egio Artal, C. Active citizenship of women and youth in Nicaragua. 2015
  - Guix, I. Building gender sensitive resilience through women's economic empowerment: Lessons learned from pastoralist women in Ethiopia. 2015
9. Simpson, J. Everyone belongs: A toolkit for applying intersectionality. 2009. Canadian Research Institute for the Advancement of Women.
10. JHPIEGO. Gender Analysis Toolkit for Health Systems. Johns Hopkins University. 2015.
11. Rosette, AS, Zhou Koval C, Ma, A& Livingston R. Race matters for women leaders: Intersectional effects on agentic deficiencies and penalties. The Leadership Quarterly; 2016. Pp.429–445.

### II. Ideas for an organizational gender analysis approach that addresses intersecting inequalities

There are several debates, including whether one or another axis of oppression/marginalization has primacy. “Intersectionality” posits that our complex identities are interconnected and cannot be examined separately. An early idea was that race and gender and class are not “additive” but rather compound and intersect each other, hence the beginning of the idea of intersectionality.^6^ Thus, our experiences are colored not only by our gender, but also by our race, class, sexuality^7^. The ideological, theoretical and methodological implications are still being addressed.

Based on research from several countries, including the two in this study, the authors nevertheless believe that the analysis of gender relations is a foremost path for understanding the organizational as well as societal contexts of women's underrepresentation in senior roles in global health organizations. That is, gender is a *key axis* of oppression and marginalization to consider in the face of the apparently ubiquitous hierarchical social differences between men and women in patriarchal societies. A more “granular” understanding gender discrimination and inequality requires a fineness of focus that might be lost to anti-categorical intersectional research, which assumes that no one axis of oppression trumps another. In any case, how gender operates in the health workforce and in organizations are still areas that are incompletely understood. Research papers are still relatively rare,^8^ suggesting that health system leaders do not have adequate information about a fundamental dimension of the diversity of experiences and needs of health workforces. For example, the research results from the Zambia and Uganda organizations presented in Appendix 2 demonstrate a constellation of unfavorable distinctions related to reproduction and reproductive roles targeting women of childbearing age, covering pregnancy, childbirth, related medical conditions, breastfeeding and family responsibilities. In combination, these appear to constitute pervasive *reproductive role discrimination* about which little is documented in health systems, and when undetected or ignored, likely results in intentional or unintentional exclusion of female workers from professional opportunity, and in abridgements of employment rights and protections. These and other organizational inequality-generating mechanisms and processes mentioned in the manuscript should be further substantiated in research in order to inform human resources and organizational development practices. Finally, as Johnson^9^ notes,
“There has been a great deal of struggle within women's movements about the relationship between patriarchy and other forms of privilege, especially those based on race, class and sexual orientation. There has also been debate over whether some forms of privilege are more important to attack first or produce more oppressive consequences than others. One way out of this conflict is to realize that patriarchy isn't problematic just because it emphasizes male dominance, but because it emphasizes dominance and control as ends (which) draw from common roots, and whatever we do to draw attention to those roots undermines them all… if we identify the core problem as any society organized around privilege, then changing that requires us to pay attention to all forms of privilege and oppression…Whether we begin by race or gender or disability status or class, if we name the problem correctly, we'll wind up going in the same general direction” (p.242).

The proposal to integrate intersecting axes of oppression and inequality in *organizational-level* gender analysis in health is new and should be a part of a global health and gender learning agenda. While we cannot lay out here the entire structure of an organizational gender analysis that addresses intersecting axes of oppression and inequality, we can address the following purposes and answer the following kinds of questions:

**Purposes**: 1) Identify exclusions, distinctions or restrictions made on the basis of socially constructed roles and norms that prevents a person from enjoying full human rights; 2)

Identify barriers and constraints to the equal chance of choosing a health occupation, developing the requisite skills and knowledge, being fairly paid, enjoying equal treatment and advancing in a career; 3) Use results for organizational learning to change the ways in which opportunities for varied roles and senior representation play out so differently based on gender; and 4) Identify common interests for the building coalitions for change within and outside the organization.

**Illustrative questions**

- What are the relations of power that constitute the root causes of gender inequality in the organization?
- What dimensions of identity and social status such as class, racial, ethnic, caste, religious and other markers create different social hierarchies and opportunities and new disadvantages in the organization?
- What are the relational differences in women's and men's
  - roles and identities
  - beliefs, perceptions, stereotypes
  - access to resources
  - legal rights and status
  - access to and exercise of power
- How do gender relations and other hierarchies affect the achievement organizational goals?
- What are the organizational gendering processes, mechanisms and structures of gender inequality regimes?
- In what ways are women targeted by violent behavior (including bullying and harassment) in the organization—what makes that violence possible or necessary?
- What aspects of organizational structure and culture affect the achievement of goals/women's opportunities for education, employment, participation and senior leadership?
- What are the large-scale patterns of inequality that can be found across the institution, such as the contrast between masculinity and femininity, the gender division of labor in the home, or the organization of sexual desire along heterosexual/homosexual lines?^10^
- What is composition and structure of the workforce? How does the organization design/structure work and arrangements for work-life reconciliation?
- What forms of discrimination and violence (e.g., sexual harassment) exist (including compound discriminations)?
- What are the main gender stereotypes (such as those related to reproductive roles, science and leadership for men and women) including possibly intersecting stereotypes (e.g., young minority woman, older man, older abled woman, etc.) to be “named” and challenged?
- How do “masculinities and femininities” play into ‘the constitution, reproduction and allocation’ of organizational power and meaning, and rewards”? How do these differ by ethnicity, race, disability, etc.?
- What policies promote equality and nondiscrimination and govern employee and management relations?
- How will proposed results of organizational development efforts affect the relative status of men and women (in different aspects of organizational life)?
